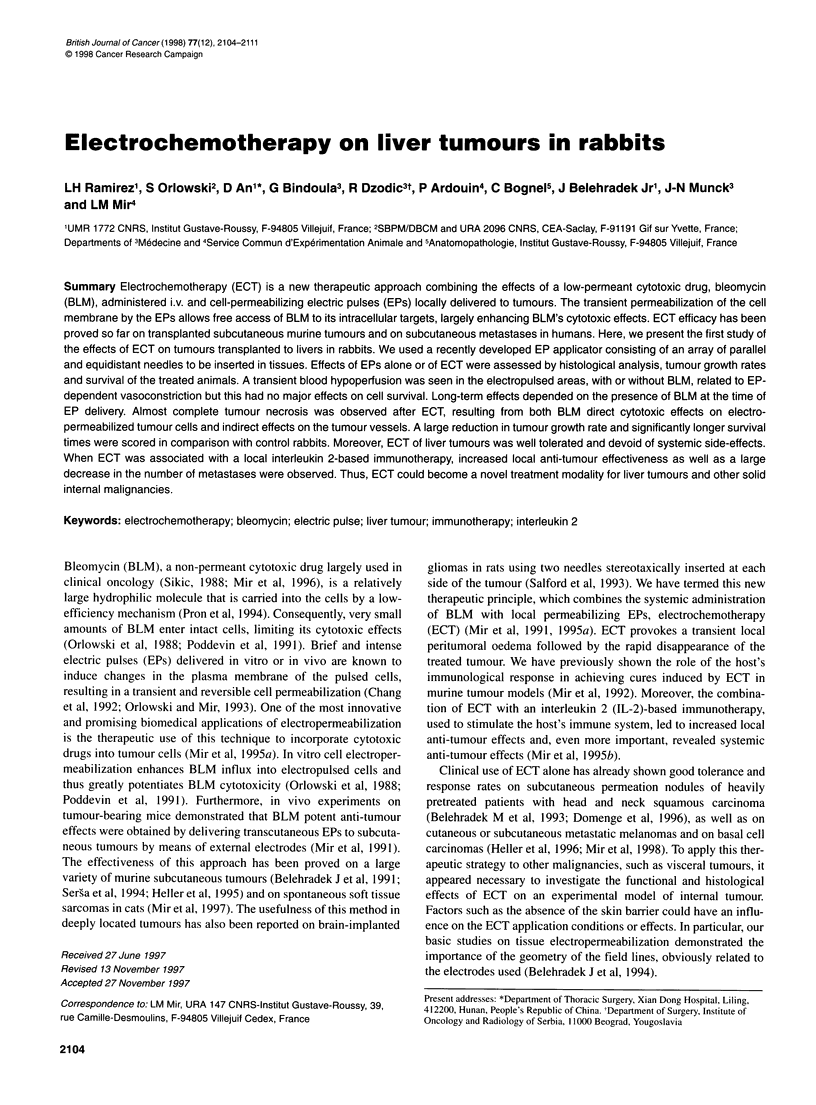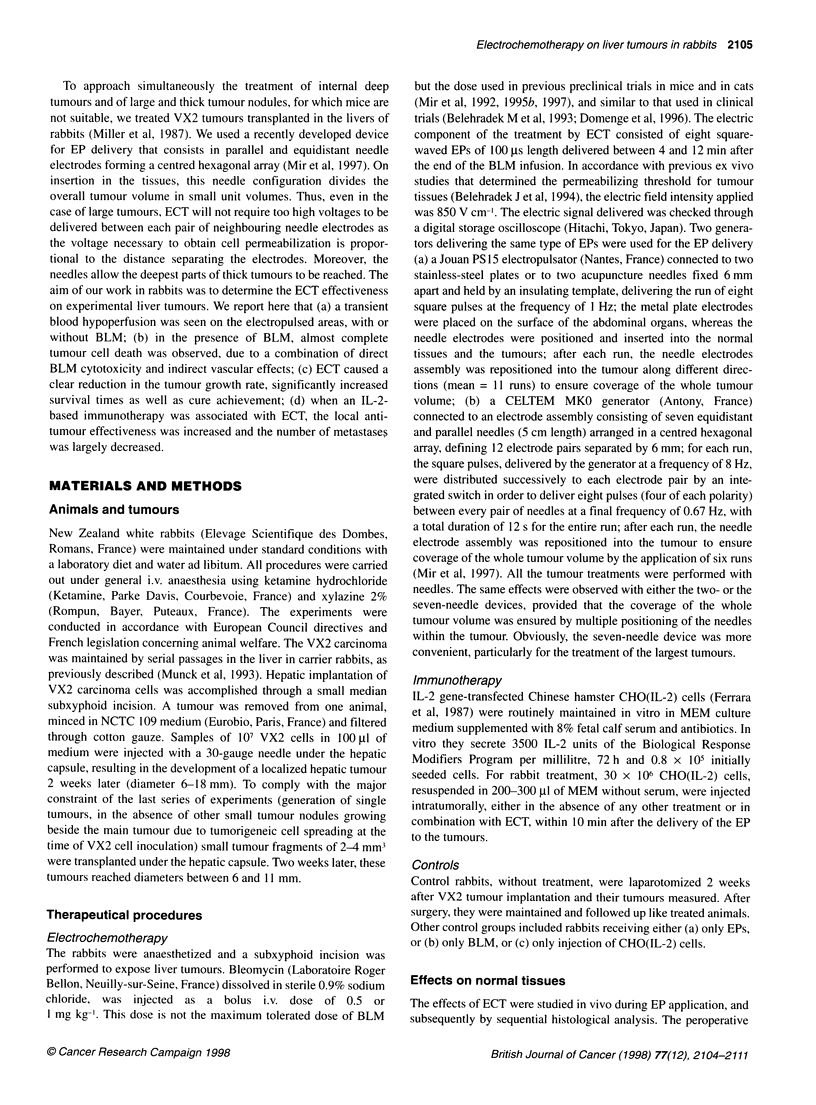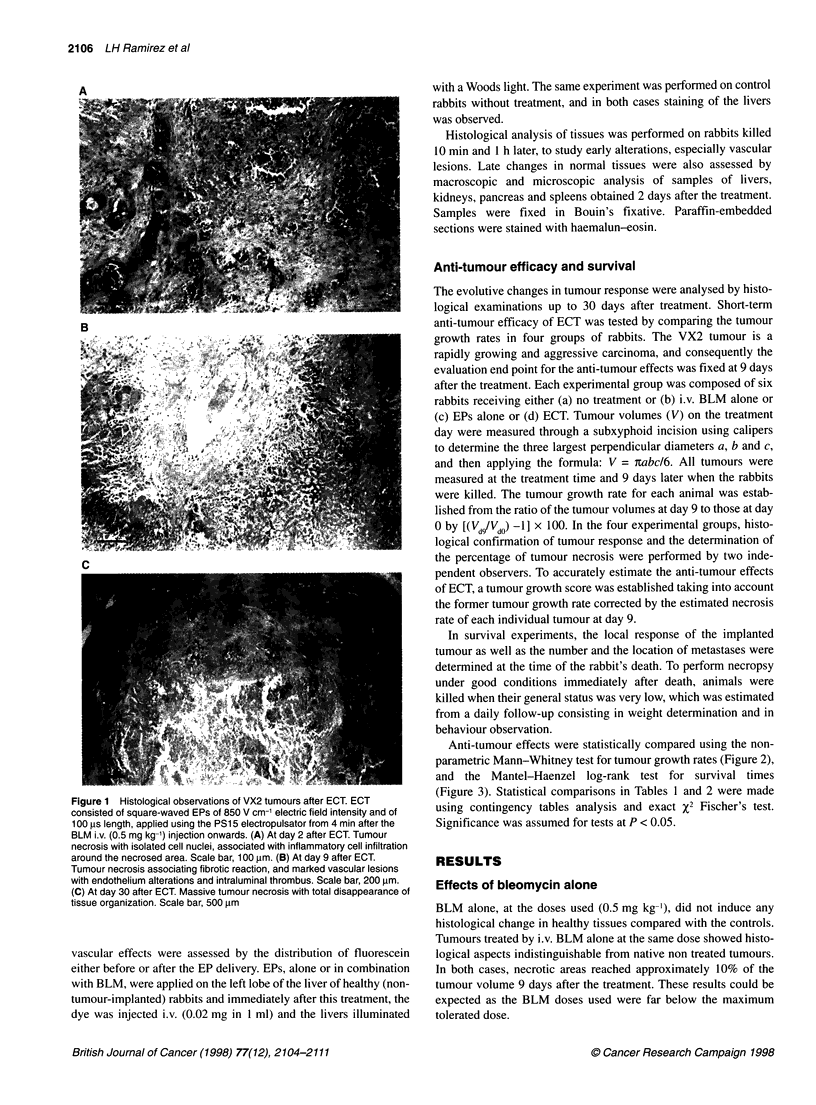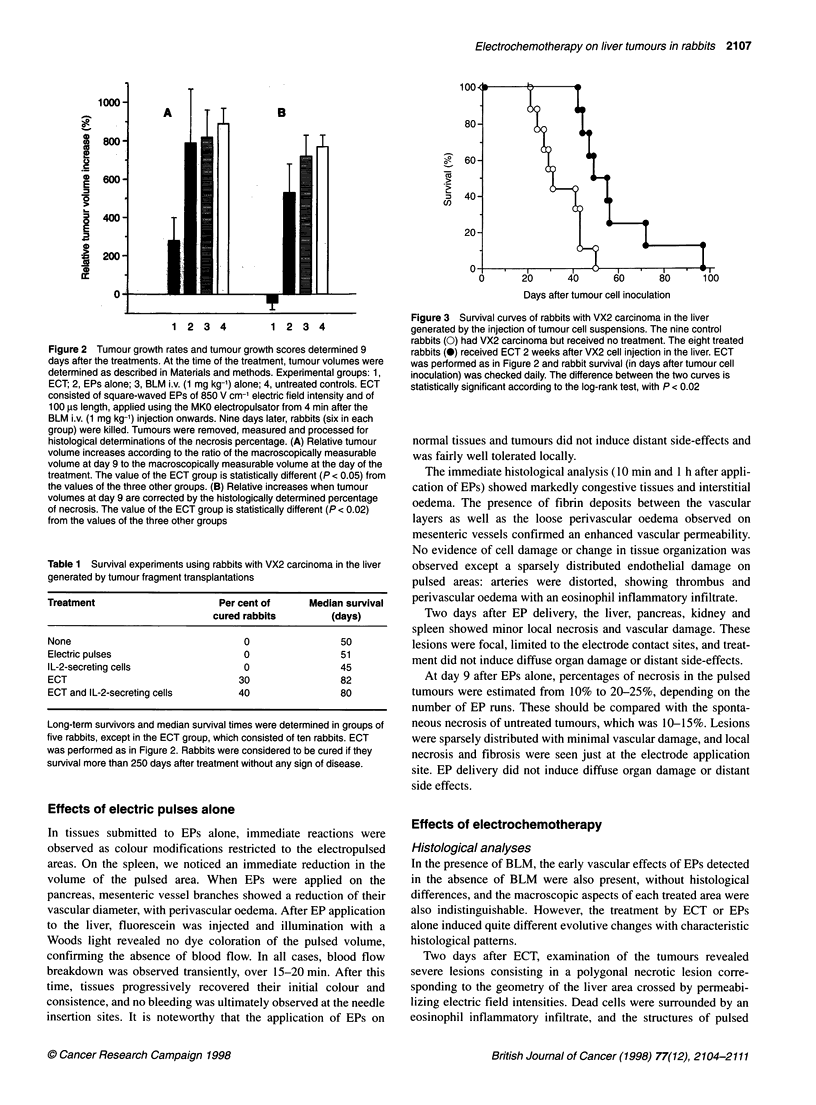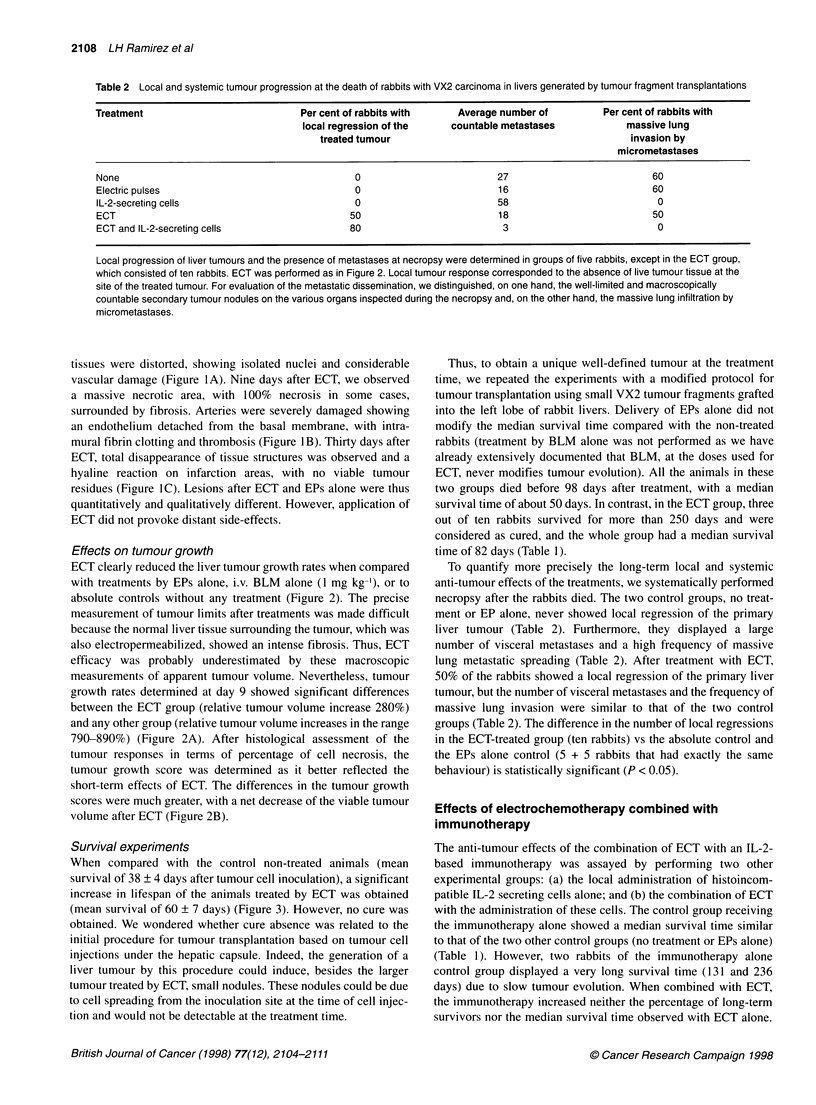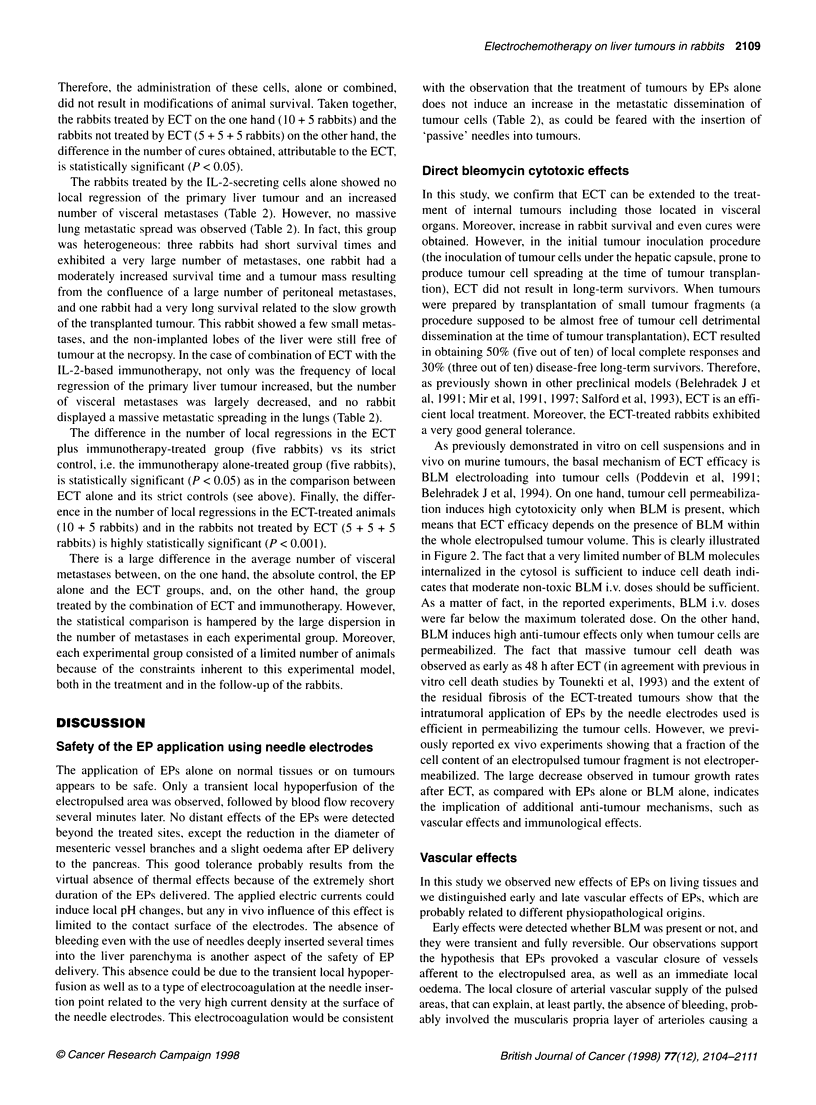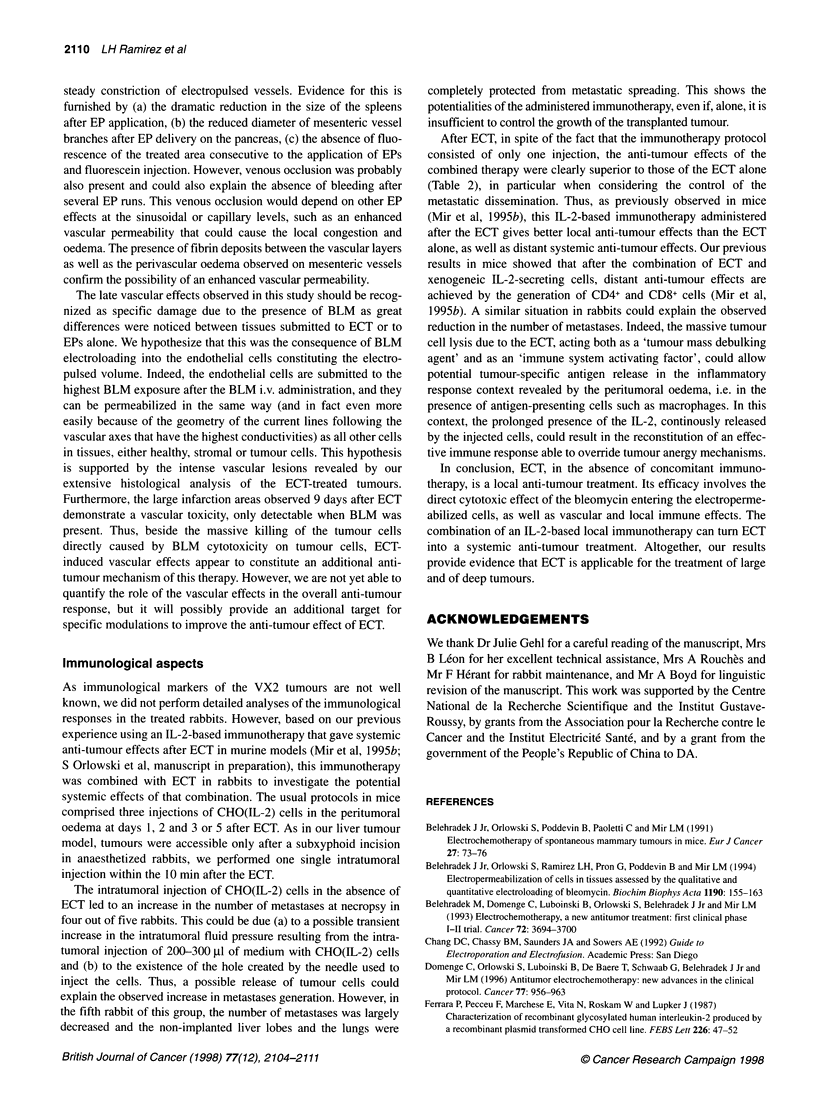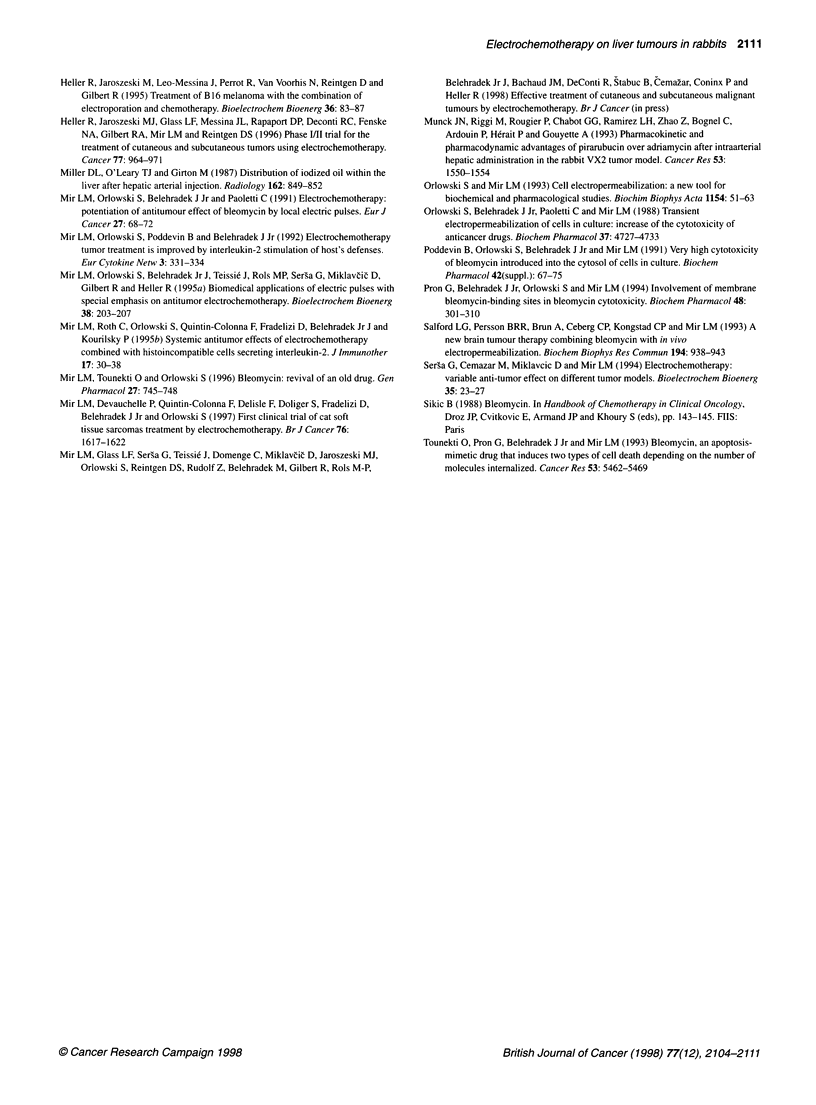# Electrochemotherapy on liver tumours in rabbits.

**DOI:** 10.1038/bjc.1998.354

**Published:** 1998-06

**Authors:** L. H. Ramirez, S. Orlowski, D. An, G. Bindoula, R. Dzodic, P. Ardouin, C. Bognel, J. Belehradek, J. N. Munck, L. M. Mir

**Affiliations:** UMR 1772 CNRS, Institut Gustave-Roussy, Villejuif, France.

## Abstract

**Images:**


British Journal of Cancer (1998) 77(12), 2104–2111
© 1998 Cancer Research Campaign

### Electrochemotherapy on liver tumours in rabbits

**LH Ramirez[1], S Orlowski[2], D An[1*], G Bindoula[3], R Dzodic[3†], P Ardouin[4], C Bognel[5], J Belehradek Jr[1], J-N Munck[3] and LM Mir[4]**

[1]UMR 1772 CNRS, Institut Gustave-Roussy, F-94805 Villejuif, France; [2]SBPM/DBCM and URA 2096 CNRS, CEA-Saclay, F-91191 Gif sur Yvette, France; Departments of [3]Médecine and [4]Service Commun d'Expérimentation Animale and [5]Anatomopathologie, Institut Gustave-Roussy, F-94805 Villejuif, France

**Summary** Electrochemotherapy (ECT) is a new therapeutic approach combining the effects of a low-permeant cytotoxic drug, bleomycin (BLM), administered i.v. and cell-permeabilizing electric pulses (EPs) locally delivered to tumours. The transient permeabilization of the cell membrane by the EPs allows free access of BLM to its intracellular targets, largely enhancing BLM's cytotoxic effects. ECT efficacy has been proved so far on transplanted subcutaneous murine tumours and on subcutaneous metastases in humans. Here, we present the first study of the effects of ECT on tumours transplanted to livers in rabbits. We used a recently developed EP applicator consisting of an array of parallel and equidistant needles to be inserted in tissues. Effects of EPs alone or of ECT were assessed by histological analysis, tumour growth rates and survival of the treated animals. A transient blood hypoperfusion was seen in the electropulsed areas, with or without BLM, related to EP-dependent vasoconstriction but this had no major effects on cell survival. Long-term effects depended on the presence of BLM at the time of EP delivery. Almost complete tumour necrosis was observed after ECT, resulting from both BLM direct cytotoxic effects on electro-permeabilized tumour cells and indirect effects on the tumour vessels. A large reduction in tumour growth rate and significantly longer survival times were scored in comparison with control rabbits. Moreover, ECT of liver tumours was well tolerated and devoid of systemic side-effects. When ECT was associated with a local interleukin 2-based immunotherapy, increased local anti-tumour effectiveness as well as a large decrease in the number of metastases were observed. Thus, ECT could become a novel treatment modality for liver tumours and other solid internal malignancies.

**Keywords:** electrochemotherapy; bleomycin; electric pulse; liver tumour; immunotherapy; interleukin 2

Bleomycin (BLM), a non-permeant cytotoxic drug largely used in clinical oncology (Sikic, 1988; Mir et al, 1996), is a relatively large hydrophilic molecule that is carried into the cells by a low-efficiency mechanism (Pron et al, 1994). Consequently, very small amounts of BLM enter intact cells, limiting its cytotoxic effects (Orlowski et al, 1988; Poddevin et al, 1991). Brief and intense electric pulses (EPs) delivered in vitro or in vivo are known to induce changes in the plasma membrane of the pulsed cells, resulting in a transient and reversible cell permeabilization (Chang et al, 1992; Orlowski and Mir, 1993). One of the most innovative and promising biomedical applications of electropermeabilization is the therapeutic use of this technique to incorporate cytotoxic drugs into tumour cells (Mir et al, 1995*a*). In vitro cell electropermeabilization enhances BLM influx into electropulsed cells and thus greatly potentiates BLM cytotoxicity (Orlowski et al, 1988; Poddevin et al, 1991). Furthermore, in vivo experiments on tumour-bearing mice demonstrated that BLM potent anti-tumour effects were obtained by delivering transcutaneous EPs to subcutaneous tumours by means of external electrodes (Mir et al, 1991). The effectiveness of this approach has been proved on a large variety of murine subcutaneous tumours (Belehradek J et al, 1991; Serša et al, 1994; Heller et al, 1995) and on spontaneous soft tissue sarcomas in cats (Mir et al, 1997). The usefulness of this method in deeply located tumours has also been reported on brain-implanted gliomas in rats using two needles stereotaxically inserted at each side of the tumour (Salford et al, 1993). We have termed this new therapeutic principle, which combines the systemic administration of BLM with local permeabilizing EPs, electrochemotherapy (ECT) (Mir et al, 1991, 1995*a*). ECT provokes a transient local peritumoral oedema followed by the rapid disappearance of the treated tumour. We have previously shown the role of the host's immunological response in achieving cures induced by ECT in murine tumour models (Mir et al, 1992). Moreover, the combination of ECT with an interleukin 2 (IL-2)-based immunotherapy, used to stimulate the host's immune system, led to increased local anti-tumour effects and, even more important, revealed systemic anti-tumour effects (Mir et al, 1995*b*).

Clinical use of ECT alone has already shown good tolerance and response rates on subcutaneous permeation nodules of heavily pretreated patients with head and neck squamous carcinoma (Belehradek M et al, 1993; Domenge et al, 1996), as well as on cutaneous or subcutaneous metastatic melanomas and on basal cell carcinomas (Heller et al, 1996; Mir et al, 1998). To apply this therapeutic strategy to other malignancies, such as visceral tumours, it appeared necessary to investigate the functional and histological effects of ECT on an experimental model of internal tumour. Factors such as the absence of the skin barrier could have an influence on the ECT application conditions or effects. In particular, our basic studies on tissue electropermeabilization demonstrated the importance of the geometry of the field lines, obviously related to the electrodes used (Belehradek J et al, 1994).

*Received 27 June 1997*
*Revised 13 November 1997*
*Accepted 27 November 1997*

*Correspondence to:* LM Mir, URA 147 CNRS-Institut Gustave-Roussy, 39, rue Camille-Desmoulins, F-94805 Villejuif Cedex, France

Present addresses: *Department of Thoracic Surgery, Xian Dong Hospital, Liling, 412200, Hunan, People's Republic of China. †Department of Surgery, Institute of Oncology and Radiology of Serbia, 11000 Beograd, Yougoslavia

To approach simultaneously the treatment of internal deep tumours and of large and thick tumour nodules, for which mice are not suitable, we treated VX2 tumours transplanted in the livers of rabbits (Miller et al, 1987). We used a recently developed device for EP delivery that consists in parallel and equidistant needle electrodes forming a centred hexagonal array (Mir et al, 1997). On insertion in the tissues, this needle configuration divides the overall tumour volume in small unit volumes. Thus, even in the case of large tumours, ECT will not require too high voltages to be delivered between each pair of neighbouring needle electrodes as the voltage necessary to obtain cell permeabilization is proportional to the distance separating the electrodes. Moreover, the needles allow the deepest parts of thick tumours to be reached. The aim of our work in rabbits was to determine the ECT effectiveness on experimental liver tumours. We report here that (a) a transient blood hypoperfusion was seen on the electropulsed areas, with or without BLM; (b) in the presence of BLM, almost complete tumour cell death was observed, due to a combination of direct BLM cytotoxicity and indirect vascular effects; (c) ECT caused a clear reduction in the tumour growth rate, significantly increased survival times as well as cure achievement; (d) when an IL-2-based immunotherapy was associated with ECT, the local anti-tumour effectiveness was increased and the number of metastases was largely decreased.

### MATERIALS AND METHODS

### Animals and tumours

New Zealand white rabbits (Elevage Scientifique des Dombes, Romans, France) were maintained under standard conditions with a laboratory diet and water ad libitum. All procedures were carried out under general i.v. anaesthesia using ketamine hydrochloride (Ketamine, Parke Davis, Courbevoie, France) and xylazine 2% (Rompun, Bayer, Puteaux, France). The experiments were conducted in accordance with European Council directives and French legislation concerning animal welfare. The VX2 carcinoma was maintained by serial passages in the liver in carrier rabbits, as previously described (Munck et al, 1993). Hepatic implantation of VX2 carcinoma cells was accomplished through a small median subxyphoid incision. A tumour was removed from one animal, minced in NCTC 109 medium (Eurobio, Paris, France) and filtered through cotton gauze. Samples of $10^7$ VX2 cells in 100 μl of medium were injected with a 30-gauge needle under the hepatic capsule, resulting in the development of a localized hepatic tumour 2 weeks later (diameter 6–18 mm). To comply with the major constraint of the last series of experiments (generation of single tumours, in the absence of other small tumour nodules growing beside the main tumour due to tumorigeneic cell spreading at the time of VX2 cell inoculation) small tumour fragments of 2–4 $mm^3$ were transplanted under the hepatic capsule. Two weeks later, these tumours reached diameters between 6 and 11 mm.

### Therapeutical procedures

#### *Electrochemotherapy*

The rabbits were anaesthetized and a subxyphoid incision was performed to expose liver tumours. Bleomycin (Laboratoire Roger Bellon, Neuilly-sur-Seine, France) dissolved in sterile 0.9% sodium chloride, was injected as a bolus i.v. dose of 0.5 or 1 mg $kg^{-1}$. This dose is not the maximum tolerated dose of BLM but the dose used in previous preclinical trials in mice and in cats (Mir et al, 1992, 1995*b*, 1997), and similar to that used in clinical trials (Belehradek M et al, 1993; Domenge et al, 1996). The electric component of the treatment by ECT consisted of eight square-waved EPs of 100 μs length delivered between 4 and 12 min after the end of the BLM infusion. In accordance with previous ex vivo studies that determined the permeabilizing threshold for tumour tissues (Belehradek J et al, 1994), the electric field intensity applied was 850 V $cm^{-1}$. The electric signal delivered was checked through a digital storage oscilloscope (Hitachi, Tokyo, Japan). Two generators delivering the same type of EPs were used for the EP delivery (a) a Jouan PS15 electropulsator (Nantes, France) connected to two stainless-steel plates or to two acupuncture needles fixed 6 mm apart and held by an insulating template, delivering the run of eight square pulses at the frequency of 1 Hz; the metal plate electrodes were placed on the surface of the abdominal organs, whereas the needle electrodes were positioned and inserted into the normal tissues and the tumours; after each run, the needle electrodes assembly was repositioned into the tumour along different directions (mean = 11 runs) to ensure coverage of the whole tumour volume; (b) a CELTEM MK0 generator (Antony, France) connected to an electrode assembly consisting of seven equidistant and parallel needles (5 cm length) arranged in a centred hexagonal array, defining 12 electrode pairs separated by 6 mm; for each run, the square pulses, delivered by the generator at a frequency of 8 Hz, were distributed successively to each electrode pair by an integrated switch in order to deliver eight pulses (four of each polarity) between every pair of needles at a final frequency of 0.67 Hz, with a total duration of 12 s for the entire run; after each run, the needle electrode assembly was repositioned into the tumour to ensure coverage of the whole tumour volume by the application of six runs (Mir et al, 1997). All the tumour treatments were performed with needles. The same effects were observed with either the two- or the seven-needle devices, provided that the coverage of the whole tumour volume was ensured by multiple positioning of the needles within the tumour. Obviously, the seven-needle device was more convenient, particularly for the treatment of the largest tumours.

#### *Immunotherapy*

IL-2 gene-transfected Chinese hamster CHO(IL-2) cells (Ferrara et al, 1987) were routinely maintained in vitro in MEM culture medium supplemented with 8% fetal calf serum and antibiotics. In vitro they secrete 3500 IL-2 units of the Biological Response Modifiers Program per millilitre, 72 h and $0.8 \times 10^5$ initially seeded cells. For rabbit treatment, $30 \times 10^6$ CHO(IL-2) cells, resuspended in 200–300 μl of MEM without serum, were injected intratumorally, either in the absence of any other treatment or in combination with ECT, within 10 min after the delivery of the EP to the tumours.

#### *Controls*

Control rabbits, without treatment, were laparotomized 2 weeks after VX2 tumour implantation and their tumours measured. After surgery, they were maintained and followed up like treated animals. Other control groups included rabbits receiving either (a) only EPs, or (b) only BLM, or (c) only injection of CHO(IL-2) cells.

### Effects on normal tissues

The effects of ECT were studied in vivo during EP application, and subsequently by sequential histological analysis. The peroperative

© Cancer Research Campaign 1998

**Figure 1** Histological observations of VX2 tumours after ECT. ECT consisted of square-waved EPs of 850 V cm$^{-1}$ electric field intensity and of 100 µs length, applied using the PS15 electropulsator from 4 min after the BLM i.v. (0.5 mg kg$^{-1}$) injection onwards. (**A**) At day 2 after ECT. Tumour necrosis with isolated cell nuclei, associated with inflammatory cell infiltration around the necrosed area. Scale bar, 100 µm. (**B**) At day 9 after ECT. Tumour necrosis associating fibrotic reaction, and marked vascular lesions with endothelium alterations and intraluminal thrombus. Scale bar, 200 µm. (**C**) At day 30 after ECT. Massive tumour necrosis with total disappearance of tissue organization. Scale bar, 500 µm vascular effects were assessed by the distribution of fluorescein either before or after the EP delivery. EPs, alone or in combination with BLM, were applied on the left lobe of the liver of healthy (non-tumour-implanted) rabbits and immediately after this treatment, the dye was injected i.v. (0.02 mg in 1 ml) and the livers illuminated with a Woods light. The same experiment was performed on control rabbits without treatment, and in both cases staining of the livers was observed.

Histological analysis of tissues was performed on rabbits killed 10 min and 1 h later, to study early alterations, especially vascular lesions. Late changes in normal tissues were also assessed by macroscopic and microscopic analysis of samples of livers, kidneys, pancreas and spleens obtained 2 days after the treatment. Samples were fixed in Bouin's fixative. Paraffin-embedded sections were stained with haemalun–eosin.

### Anti-tumour efficacy and survival

The evolutive changes in tumour response were analysed by histological examinations up to 30 days after treatment. Short-term anti-tumour efficacy of ECT was tested by comparing the tumour growth rates in four groups of rabbits. The VX2 tumour is a rapidly growing and aggressive carcinoma, and consequently the evaluation end point for the anti-tumour effects was fixed at 9 days after the treatment. Each experimental group was composed of six rabbits receiving either (a) no treatment or (b) i.v. BLM alone or (c) EPs alone or (d) ECT. Tumour volumes ($V$) on the treatment day were measured through a subxyphoid incision using calipers to determine the three largest perpendicular diameters $a$, $b$ and $c$, and then applying the formula: $V = \pi abc/6$. All tumours were measured at the treatment time and 9 days later when the rabbits were killed. The tumour growth rate for each animal was established from the ratio of the tumour volumes at day 9 to those at day 0 by $[(V_{d9}/V_{d0}) - 1] \times 100$. In the four experimental groups, histological confirmation of tumour response and the determination of the percentage of tumour necrosis were performed by two independent observers. To accurately estimate the anti-tumour effects of ECT, a tumour growth score was established taking into account the former tumour growth rate corrected by the estimated necrosis rate of each individual tumour at day 9.

In survival experiments, the local response of the implanted tumour as well as the number and the location of metastases were determined at the time of the rabbit's death. To perform necropsy under good conditions immediately after death, animals were killed when their general status was very low, which was estimated from a daily follow-up consisting in weight determination and in behaviour observation.

Anti-tumour effects were statistically compared using the non-parametric Mann–Whitney test for tumour growth rates (Figure 2), and the Mantel–Haenzel log-rank test for survival times (Figure 3). Statistical comparisons in Tables 1 and 2 were made using contingency tables analysis and exact $\chi^2$ Fischer's test. Significance was assumed for tests at $P < 0.05$.

### RESULTS

### Effects of bleomycin alone

BLM alone, at the doses used (0.5 mg kg$^{-1}$), did not induce any histological change in healthy tissues compared with the controls. Tumours treated by i.v. BLM alone at the same dose showed histological aspects indistinguishable from native non treated tumours. In both cases, necrotic areas reached approximately 10% of the tumour volume 9 days after the treatment. These results could be expected as the BLM doses used were far below the maximum tolerated dose.

© Cancer Research Campaign 1998

**Figure 2** Tumour growth rates and tumour growth scores determined 9 days after the treatments. At the time of the treatment, tumour volumes were determined as described in Materials and methods. Experimental groups: 1, ECT; 2, EPs alone; 3, BLM i.v. (1 mg kg$^{-1}$) alone; 4, untreated controls. ECT consisted of square-waved EPs of 850 V cm$^{-1}$ electric field intensity and of 100 μs length, applied using the MKO electropulsator from 4 min after the BLM i.v. (1 mg kg$^{-1}$) injection onwards. Nine days later, rabbits (six in each group) were killed. Tumours were removed, measured and processed for histological determinations of the necrosis percentage. (**A**) Relative tumour volume increases according to the ratio of the macroscopically measurable volume at day 9 to the macroscopically measurable volume at the day of the treatment. The value of the ECT group is statistically different ($P < 0.05$) from the values of the three other groups. (**B**) Relative increases when tumour volumes at day 9 are corrected by the histologically determined percentage of necrosis. The value of the ECT group is statistically different ($P < 0.02$) from the values of the three other groups

**Table 1** Survival experiments using rabbits with VX2 carcinoma in the liver generated by tumour fragment transplantations

| Treatment | Per cent of cured rabbits | Median survival (days) |
|---|---|---|
| None | 0 | 50 |
| Electric pulses | 0 | 51 |
| IL-2-secreting cells | 0 | 45 |
| ECT | 30 | 82 |
| ECT and IL-2-secreting cells | 40 | 80 |

Long-term survivors and median survival times were determined in groups of five rabbits, except in the ECT group, which consisted of ten rabbits. ECT was performed as in Figure 2. Rabbits were considered to be cured if they survival more than 250 days after treatment without any sign of disease.

### Effects of electric pulses alone

In tissues submitted to EPs alone, immediate reactions were observed as colour modifications restricted to the electropulsed areas. On the spleen, we noticed an immediate reduction in the volume of the pulsed area. When EPs were applied on the pancreas, mesenteric vessel branches showed a reduction of their vascular diameter, with perivascular oedema. After EP application to the liver, fluorescein was injected and illumination with a Woods light revealed no dye coloration of the pulsed volume, confirming the absence of blood flow. In all cases, blood flow breakdown was observed transiently, over 15–20 min. After this time, tissues progressively recovered their initial colour and consistence, and no bleeding was ultimately observed at the needle insertion sites. It is noteworthy that the application of EPs on normal tissues and tumours did not induce distant side-effects and was fairly well tolerated locally.

**Figure 3** Survival curves of rabbits with VX2 carcinoma in the liver generated by the injection of tumour cell suspensions. The nine control rabbits (○) had VX2 carcinoma but received no treatment. The eight treated rabbits (●) received ECT 2 weeks after VX2 cell injection in the liver. ECT was performed as in Figure 2 and rabbit survival (in days after tumour cell inoculation) was checked daily. The difference between the two curves is statistically significant according to the log-rank test, with $P < 0.02$

The immediate histological analysis (10 min and 1 h after application of EPs) showed markedly congestive tissues and interstitial oedema. The presence of fibrin deposits between the vascular layers as well as the loose perivascular oedema observed on mesenteric vessels confirmed an enhanced vascular permeability. No evidence of cell damage or change in tissue organization was observed except a sparsely distributed endothelial damage on pulsed areas: arteries were distorted, showing thrombus and perivascular oedema with an eosinophil inflammatory infiltrate.

Two days after EP delivery, the liver, pancreas, kidney and spleen showed minor local necrosis and vascular damage. These lesions were focal, limited to the electrode contact sites, and treatment did not induce diffuse organ damage or distant side-effects.

At day 9 after EPs alone, percentages of necrosis in the pulsed tumours were estimated from 10% to 20–25%, depending on the number of EP runs. These should be compared with the spontaneous necrosis of untreated tumours, which was 10–15%. Lesions were sparsely distributed with minimal vascular damage, and local necrosis and fibrosis were seen just at the electrode application site. EP delivery did not induce diffuse organ damage or distant side effects.

### Effects of electrochemotherapy

### *Histological analyses*

In the presence of BLM, the early vascular effects of EPs detected in the absence of BLM were also present, without histological differences, and the macroscopic aspects of each treated area were also indistinguishable. However, the treatment by ECT or EPs alone induced quite different evolutive changes with characteristic histological patterns.

Two days after ECT, examination of the tumours revealed severe lesions consisting in a polygonal necrotic lesion corresponding to the geometry of the liver area crossed by permeabilizing electric field intensities. Dead cells were surrounded by an eosinophil inflammatory infiltrate, and the structures of pulsed

**Table 2** Local and systemic tumour progression at the death of rabbits with VX2 carcinoma in livers generated by tumour fragment transplantations

| Treatment | Per cent of rabbits with local regression of the treated tumour | Average number of countable metastases | Per cent of rabbits with massive lung invasion by micrometastases |
|---|---|---|---|
| None | 0 | 27 | 60 |
| Electric pulses | 0 | 16 | 60 |
| IL-2-secreting cells | 0 | 58 | 0 |
| ECT | 50 | 18 | 50 |
| ECT and IL-2-secreting cells | 80 | 3 | 0 |

Local progression of liver tumours and the presence of metastases at necropsy were determined in groups of five rabbits, except in the ECT group, which consisted of ten rabbits. ECT was performed as in Figure 2. Local tumour response corresponded to the absence of live tumour tissue at the site of the treated tumour. For evaluation of the metastatic dissemination, we distinguished, on one hand, the well-limited and macroscopically countable secondary tumour nodules on the various organs inspected during the necropsy and, on the other hand, the massive lung infiltration by micrometastases.

tissues were distorted, showing isolated nuclei and considerable vascular damage (Figure 1A). Nine days after ECT, we observed a massive necrotic area, with 100% necrosis in some cases, surrounded by fibrosis. Arteries were severely damaged showing an endothelium detached from the basal membrane, with intramural fibrin clotting and thrombosis (Figure 1B). Thirty days after ECT, total disappearance of tissue structures was observed and a hyaline reaction on infarction areas, with no viable tumour residues (Figure 1C). Lesions after ECT and EPs alone were thus quantitatively and qualitatively different. However, application of ECT did not provoke distant side-effects.

### *Effects on tumour growth*

ECT clearly reduced the liver tumour growth rates when compared with treatments by EPs alone, i.v. BLM alone (1 mg $kg^{-1}$), or to absolute controls without any treatment (Figure 2). The precise measurement of tumour limits after treatments was made difficult because the normal liver tissue surrounding the tumour, which was also electropermeabilized, showed an intense fibrosis. Thus, ECT efficacy was probably underestimated by these macroscopic measurements of apparent tumour volume. Nevertheless, tumour growth rates determined at day 9 showed significant differences between the ECT group (relative tumour volume increase 280%) and any other group (relative tumour volume increases in the range 790–890%) (Figure 2A). After histological assessment of the tumour responses in terms of percentage of cell necrosis, the tumour growth score was determined as it better reflected the short-term effects of ECT. The differences in the tumour growth scores were much greater, with a net decrease of the viable tumour volume after ECT (Figure 2B).

### *Survival experiments*

When compared with the control non-treated animals (mean survival of 38 ± 4 days after tumour cell inoculation), a significant increase in lifespan of the animals treated by ECT was obtained (mean survival of 60 ± 7 days) (Figure 3). However, no cure was obtained. We wondered whether cure absence was related to the initial procedure for tumour transplantation based on tumour cell injections under the hepatic capsule. Indeed, the generation of a liver tumour by this procedure could induce, besides the larger tumour treated by ECT, small nodules. These nodules could be due to cell spreading from the inoculation site at the time of cell injection and would not be detectable at the treatment time.

Thus, to obtain a unique well-defined tumour at the treatment time, we repeated the experiments with a modified protocol for tumour transplantation using small VX2 tumour fragments grafted into the left lobe of rabbit livers. Delivery of EPs alone did not modify the median survival time compared with the non-treated rabbits (treatment by BLM alone was not performed as we have already extensively documented that BLM, at the doses used for ECT, never modifies tumour evolution). All the animals in these two groups died before 98 days after treatment, with a median survival time of about 50 days. In contrast, in the ECT group, three out of ten rabbits survived for more than 250 days and were considered as cured, and the whole group had a median survival time of 82 days (Table 1).

To quantify more precisely the long-term local and systemic anti-tumour effects of the treatments, we systematically performed necropsy after the rabbits died. The two control groups, no treatment or EP alone, never showed local regression of the primary liver tumour (Table 2). Furthermore, they displayed a large number of visceral metastases and a high frequency of massive lung metastatic spreading (Table 2). After treatment with ECT, 50% of the rabbits showed a local regression of the primary liver tumour, but the number of visceral metastases and the frequency of massive lung invasion were similar to that of the two control groups (Table 2). The difference in the number of local regressions in the ECT-treated group (ten rabbits) vs the absolute control and the EPs alone control (5 + 5 rabbits that had exactly the same behaviour) is statistically significant ($P < 0.05$).

### Effects of electrochemotherapy combined with immunotherapy

The anti-tumour effects of the combination of ECT with an IL-2-based immunotherapy was assayed by performing two other experimental groups: (a) the local administration of histoincompatible IL-2 secreting cells alone; and (b) the combination of ECT with the administration of these cells. The control group receiving the immunotherapy alone showed a median survival time similar to that of the two other control groups (no treatment or EPs alone) (Table 1). However, two rabbits of the immunotherapy alone control group displayed a very long survival time (131 and 236 days) due to slow tumour evolution. When combined with ECT, the immunotherapy increased neither the percentage of long-term survivors nor the median survival time observed with ECT alone.

*© Cancer Research Campaign 1998*

Therefore, the administration of these cells, alone or combined, did not result in modifications of animal survival. Taken together, the rabbits treated by ECT on the one hand (10 + 5 rabbits) and the rabbits not treated by ECT (5 + 5 + 5 rabbits) on the other hand, the difference in the number of cures obtained, attributable to the ECT, is statistically significant ($P < 0.05$).

The rabbits treated by the IL-2-secreting cells alone showed no local regression of the primary liver tumour and an increased number of visceral metastases (Table 2). However, no massive lung metastatic spread was observed (Table 2). In fact, this group was heterogeneous: three rabbits had short survival times and exhibited a very large number of metastases, one rabbit had a moderately increased survival time and a tumour mass resulting from the confluence of a large number of peritoneal metastases, and one rabbit had a very long survival related to the slow growth of the transplanted tumour. This rabbit showed a few small metastases, and the non-implanted lobes of the liver were still free of tumour at the necropsy. In the case of combination of ECT with the IL-2-based immunotherapy, not only was the frequency of local regression of the primary liver tumour increased, but the number of visceral metastases was largely decreased, and no rabbit displayed a massive metastatic spreading in the lungs (Table 2).

The difference in the number of local regressions in the ECT plus immunotherapy-treated group (five rabbits) vs its strict control, i.e. the immunotherapy alone-treated group (five rabbits), is statistically significant ($P < 0.05$) as in the comparison between ECT alone and its strict controls (see above). Finally, the difference in the number of local regressions in the ECT-treated animals (10 + 5 rabbits) and in the rabbits not treated by ECT (5 + 5 + 5 rabbits) is highly statistically significant ($P < 0.001$).

There is a large difference in the average number of visceral metastases between, on the one hand, the absolute control, the EP alone and the ECT groups, and, on the other hand, the group treated by the combination of ECT and immunotherapy. However, the statistical comparison is hampered by the large dispersion in the number of metastases in each experimental group. Moreover, each experimental group consisted of a limited number of animals because of the constraints inherent to this experimental model, both in the treatment and in the follow-up of the rabbits.

### DISCUSSION

### Safety of the EP application using needle electrodes

The application of EPs alone on normal tissues or on tumours appears to be safe. Only a transient local hypoperfusion of the electropulsed area was observed, followed by blood flow recovery several minutes later. No distant effects of the EPs were detected beyond the treated sites, except the reduction in the diameter of mesenteric vessel branches and a slight oedema after EP delivery to the pancreas. This good tolerance probably results from the virtual absence of thermal effects because of the extremely short duration of the EPs delivered. The applied electric currents could induce local pH changes, but any in vivo influence of this effect is limited to the contact surface of the electrodes. The absence of bleeding even with the use of needles deeply inserted several times into the liver parenchyma is another aspect of the safety of EP delivery. This absence could be due to the transient local hypoperfusion as well as to a type of electrocoagulation at the needle insertion point related to the very high current density at the surface of the needle electrodes. This electrocoagulation would be consistent with the observation that the treatment of tumours by EPs alone does not induce an increase in the metastatic dissemination of tumour cells (Table 2), as could be feared with the insertion of 'passive' needles into tumours.

### Direct bleomycin cytotoxic effects

In this study, we confirm that ECT can be extended to the treatment of internal tumours including those located in visceral organs. Moreover, increase in rabbit survival and even cures were obtained. However, in the initial tumour inoculation procedure (the inoculation of tumour cells under the hepatic capsule, prone to produce tumour cell spreading at the time of tumour transplantation), ECT did not result in long-term survivors. When tumours were prepared by transplantation of small tumour fragments (a procedure supposed to be almost free of tumour cell detrimental dissemination at the time of tumour transplantation), ECT resulted in obtaining 50% (five out of ten) of local complete responses and 30% (three out of ten) disease-free long-term survivors. Therefore, as previously shown in other preclinical models (Belehradek J et al, 1991; Mir et al, 1991, 1997; Salford et al, 1993), ECT is an efficient local treatment. Moreover, the ECT-treated rabbits exhibited a very good general tolerance.

As previously demonstrated in vitro on cell suspensions and in vivo on murine tumours, the basal mechanism of ECT efficacy is BLM electroloading into tumour cells (Poddevin et al, 1991; Belehradek J et al, 1994). On one hand, tumour cell permeabilization induces high cytotoxicity only when BLM is present, which means that ECT efficacy depends on the presence of BLM within the whole electropulsed tumour volume. This is clearly illustrated in Figure 2. The fact that a very limited number of BLM molecules internalized in the cytosol is sufficient to induce cell death indicates that moderate non-toxic BLM i.v. doses should be sufficient. As a matter of fact, in the reported experiments, BLM i.v. doses were far below the maximum tolerated dose. On the other hand, BLM induces high anti-tumour effects only when tumour cells are permeabilized. The fact that massive tumour cell death was observed as early as 48 h after ECT (in agreement with previous in vitro cell death studies by Tounekti et al, 1993) and the extent of the residual fibrosis of the ECT-treated tumours show that the intratumoral application of EPs by the needle electrodes used is efficient in permeabilizing the tumour cells. However, we previously reported ex vivo experiments showing that a fraction of the cell content of an electropulsed tumour fragment is not electropermeabilized. The large decrease observed in tumour growth rates after ECT, as compared with EPs alone or BLM alone, indicates the implication of additional anti-tumour mechanisms, such as vascular effects and immunological effects.

### Vascular effects

In this study we observed new effects of EPs on living tissues and we distinguished early and late vascular effects of EPs, which are probably related to different physiopathological origins.

Early effects were detected whether BLM was present or not, and they were transient and fully reversible. Our observations support the hypothesis that EPs provoked a vascular closure of vessels afferent to the electropulsed area, as well as an immediate local oedema. The local closure of arterial vascular supply of the pulsed areas, that can explain, at least partly, the absence of bleeding, probably involved the muscularis propria layer of arterioles causing a

© Cancer Research Campaign 1998 

steady constriction of electropulsed vessels. Evidence for this is furnished by (a) the dramatic reduction in the size of the spleens after EP application, (b) the reduced diameter of mesenteric vessel branches after EP delivery on the pancreas, (c) the absence of fluorescence of the treated area consecutive to the application of EPs and fluorescein injection. However, venous occlusion was probably also present and could also explain the absence of bleeding after several EP runs. This venous occlusion would depend on other EP effects at the sinusoidal or capillary levels, such as an enhanced vascular permeability that could cause the local congestion and oedema. The presence of fibrin deposits between the vascular layers as well as the perivascular oedema observed on mesenteric vessels confirm the possibility of an enhanced vascular permeability.

The late vascular effects observed in this study should be recognized as specific damage due to the presence of BLM as great differences were noticed between tissues submitted to ECT or to EPs alone. We hypothesize that this was the consequence of BLM electroloading into the endothelial cells constituting the electropulsed volume. Indeed, the endothelial cells are submitted to the highest BLM exposure after the BLM i.v. administration, and they can be permeabilized in the same way (and in fact even more easily because of the geometry of the current lines following the vascular axes that have the highest conductivities) as all other cells in tissues, either healthy, stromal or tumour cells. This hypothesis is supported by the intense vascular lesions revealed by our extensive histological analysis of the ECT-treated tumours. Furthermore, the large infarction areas observed 9 days after ECT demonstrate a vascular toxicity, only detectable when BLM was present. Thus, beside the massive killing of the tumour cells directly caused by BLM cytotoxicity on tumour cells, ECT-induced vascular effects appear to constitute an additional anti-tumour mechanism of this therapy. However, we are not yet able to quantify the role of the vascular effects in the overall anti-tumour response, but it will possibly provide an additional target for specific modulations to improve the anti-tumour effect of ECT.

### Immunological aspects

As immunological markers of the VX2 tumours are not well known, we did not perform detailed analyses of the immunological responses in the treated rabbits. However, based on our previous experience using an IL-2-based immunotherapy that gave systemic anti-tumour effects after ECT in murine models (Mir et al, 1995*b*; S Orlowski et al, manuscript in preparation), this immunotherapy was combined with ECT in rabbits to investigate the potential systemic effects of that combination. The usual protocols in mice comprised three injections of CHO(IL-2) cells in the peritumoral oedema at days 1, 2 and 3 or 5 after ECT. As in our liver tumour model, tumours were accessible only after a subxyphoid incision in anaesthetized rabbits, we performed one single intratumoral injection within the 10 min after the ECT.

The intratumoral injection of CHO(IL-2) cells in the absence of ECT led to an increase in the number of metastases at necropsy in four out of five rabbits. This could be due (a) to a possible transient increase in the intratumoral fluid pressure resulting from the intratumoral injection of 200–300 μl of medium with CHO(IL-2) cells and (b) to the existence of the hole created by the needle used to inject the cells. Thus, a possible release of tumour cells could explain the observed increase in metastases generation. However, in the fifth rabbit of this group, the number of metastases was largely decreased and the non-implanted liver lobes and the lungs were completely protected from metastatic spreading. This shows the potentialities of the administered immunotherapy, even if, alone, it is insufficient to control the growth of the transplanted tumour.

After ECT, in spite of the fact that the immunotherapy protocol consisted of only one injection, the anti-tumour effects of the combined therapy were clearly superior to those of the ECT alone (Table 2), in particular when considering the control of the metastatic dissemination. Thus, as previously observed in mice (Mir et al, 1995*b*), this IL-2-based immunotherapy administered after the ECT gives better local anti-tumour effects than the ECT alone, as well as distant systemic anti-tumour effects. Our previous results in mice showed that after the combination of ECT and xenogeneic IL-2-secreting cells, distant anti-tumour effects are achieved by the generation of $CD4^+$ and $CD8^+$ cells (Mir et al, 1995*b*). A similar situation in rabbits could explain the observed reduction in the number of metastases. Indeed, the massive tumour cell lysis due to the ECT, acting both as a 'tumour mass debulking agent' and as an 'immune system activating factor', could allow potential tumour-specific antigen release in the inflammatory response context revealed by the peritumoral oedema, i.e. in the presence of antigen-presenting cells such as macrophages. In this context, the prolonged presence of the IL-2, continously released by the injected cells, could result in the reconstitution of an effective immune response able to override tumour anergy mechanisms.

In conclusion, ECT, in the absence of concomitant immunotherapy, is a local anti-tumour treatment. Its efficacy involves the direct cytotoxic effect of the bleomycin entering the electropermeabilized cells, as well as vascular and local immune effects. The combination of an IL-2-based local immunotherapy can turn ECT into a systemic anti-tumour treatment. Altogether, our results provide evidence that ECT is applicable for the treatment of large and of deep tumours.

### ACKNOWLEDGEMENTS

We thank Dr Julie Gehl for a careful reading of the manuscript, Mrs B Léon for her excellent technical assistance, Mrs A Rouchès and Mr F Hérant for rabbit maintenance, and Mr A Boyd for linguistic revision of the manuscript. This work was supported by the Centre National de la Recherche Scientifique and the Institut Gustave-Roussy, by grants from the Association pour la Recherche contre le Cancer and the Institut Electricité Santé, and by a grant from the government of the People's Republic of China to DA.

### REFERENCES

Belehradek J Jr, Orlowski S, Poddevin B, Paoletti C and Mir LM (1991) Electrochemotherapy of spontaneous mammary tumours in mice. *Eur J Cancer* **27**: 73–76

Belehradek J Jr, Orlowski S, Ramirez LH, Pron G, Poddevin B and Mir LM (1994) Electropermeabilization of cells in tissues assessed by the qualitative and quantitative electroloading of bleomycin. *Biochim Biophys Acta* **1190**: 155–163

Belehradek M, Domenge C, Luboinski B, Orlowski S, Belehradek J Jr and Mir LM (1993) Electrochemotherapy, a new antitumor treatment: first clinical phase I–II trial. *Cancer* **72**: 3694–3700

Chang DC, Chassy BM, Saunders JA and Sowers AE (1992) *Guide to Electroporation and Electrofusion*. Academic Press: San Diego

Domenge C, Orlowski S, Luboinski B, De Baere T, Schwaab G, Belehradek J Jr and Mir LM (1996) Antitumor electrochemotherapy: new advances in the clinical protocol. *Cancer* **77**: 956–963

Ferrara P, Pecceu F, Marchese E, Vita N, Roskam W and Lupker J (1987) Characterization of recombinant glycosylated human interleukin-2 produced by a recombinant plasmid transformed CHO cell line. *FEBS Lett* **226**: 47–52

*© Cancer Research Campaign 1998*

Heller R, Jaroszeski M, Leo-Messina J, Perrot R, Van Voorhis N, Reintgen D and Gilbert R (1995) Treatment of B16 melanoma with the combination of electroporation and chemotherapy. *Bioelectrochem Bioenerg* **36**: 83–87

Heller R, Jaroszeski MJ, Glass LF, Messina JL, Rapaport DP, Deconti RC, Fenske NA, Gilbert RA, Mir LM and Reintgen DS (1996) Phase I/II trial for the treatment of cutaneous and subcutaneous tumors using electrochemotherapy. *Cancer* **77**: 964–971

Miller DL, O'Leary TJ and Girton M (1987) Distribution of iodized oil within the liver after hepatic arterial injection. *Radiology* **162**: 849–852

Mir LM, Orlowski S, Belehradek J Jr and Paoletti C (1991) Electrochemotherapy: potentiation of antitumour effect of bleomycin by local electric pulses. *Eur J Cancer* **27**: 68–72

Mir LM, Orlowski S, Poddevin B and Belehradek J Jr (1992) Electrochemotherapy tumor treatment is improved by interleukin-2 stimulation of host's defenses. *Eur Cytokine Netw* **3**: 331–334

Mir LM, Orlowski S, Belehradek Jr J, Teissié J, Rols MP, Serša G, Miklavčič D, Gilbert R and Heller R (1995*a*) Biomedical applications of electric pulses with special emphasis on antitumor electrochemotherapy. *Bioelectrochem Bioenerg* **38**: 203–207

Mir LM, Roth C, Orlowski S, Quintin-Colonna F, Fradelizi D, Belehradek Jr J and Kourilsky P (1995*b*) Systemic antitumor effects of electrochemotherapy combined with histoincompatible cells secreting interleukin-2. *J Immunother* **17**: 30–38

Mir LM, Tounekti O and Orlowski S (1996) Bleomycin: revival of an old drug. *Gen Pharmacol* **27**: 745–748

Mir LM, Devauchelle P, Quintin-Colonna F, Delisle F, Doliger S, Fradelizi D, Belehradek J Jr and Orlowski S (1997) First clinical trial of cat soft tissue sarcomas treatment by electrochemotherapy. *Br J Cancer* **76**: 1617–1622

Mir LM, Glass LF, Serša G, Teissié J, Domenge C, Miklavčič D, Jaroszeski MJ, Orlowski S, Reintgen DS, Rudolf Z, Belehradek M, Gilbert R, Rols M-P, Belehradek Jr J, Bachaud JM, DeConti R, Štabuc B, Čemažar, Coninx P and Heller R (1998) Effective treatment of cutaneous and subcutaneous malignant tumours by electrochemotherapy. *Br J Cancer* (in press)

Munck JN, Riggi M, Rougier P, Chabot GG, Ramirez LH, Zhao Z, Bognel C, Ardouin P, Hérait P and Gouyette A (1993) Pharmacokinetic and pharmacodynamic advantages of pirarubicin over adriamycin after intraarterial hepatic administration in the rabbit VX2 tumor model. *Cancer Res* **53**: 1550–1554

Orlowski S and Mir LM (1993) Cell electropermeabilization: a new tool for biochemical and pharmacological studies. *Biochim Biophys Acta* **1154**: 51–63

Orlowski S, Belehradek J Jr, Paoletti C and Mir LM (1988) Transient electropermeabilization of cells in culture: increase of the cytotoxicity of anticancer drugs. *Biochem Pharmacol* **37**: 4727–4733

Poddevin B, Orlowski S, Belehradek J Jr and Mir LM (1991) Very high cytotoxicity of bleomycin introduced into the cytosol of cells in culture. *Biochem Pharmacol* **42**(suppl.): 67–75

Pron G, Belehradek J Jr, Orlowski S and Mir LM (1994) Involvement of membrane bleomycin-binding sites in bleomycin cytotoxicity. *Biochem Pharmacol* **48**: 301–310

Salford LG, Persson BRR, Brun A, Ceberg CP, Kongstad CP and Mir LM (1993) A new brain tumour therapy combining bleomycin with *in vivo* electropermeabilization. *Biochem Biophys Res Commun* **194**: 938–943

Serša G, Cemazar M, Miklavcic D and Mir LM (1994) Electrochemotherapy: variable anti-tumor effect on different tumor models. *Bioelectrochem Bioenerg* **35**: 23–27

Sikic B (1988) Bleomycin. In *Handbook of Chemotherapy in Clinical Oncology*, Droz JP, Cvitkovic E, Armand JP and Khoury S (eds), pp. 143–145. FIIS: Paris

Tounekti O, Pron G, Belehradek J Jr and Mir LM (1993) Bleomycin, an apoptosis-mimetic drug that induces two types of cell death depending on the number of molecules internalized. *Cancer Res* **53**: 5462–5469

© Cancer Research Campaign 1998